# Supplementary figures and images for: Influence of scanning plane on Human Spinal Cord functional Magnetic Resonance echo planar imaging
Source: PLoS One. 2025 May 12;20(5):e0320188. doi: 10.1371/journal.pone.0320188 (PMC12068702; doi:10.1371/journal.pone.0320188)

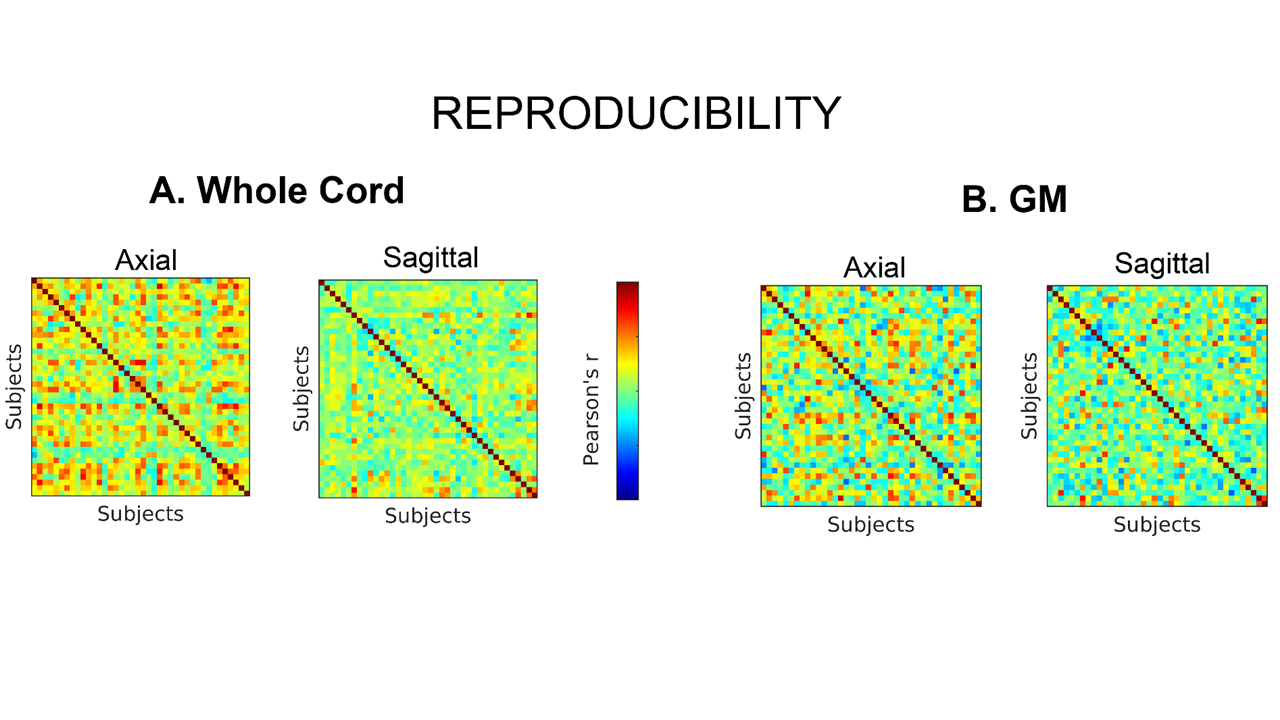

Supplement: S1 Fig — Representative tSNR maps from a healthy subject before and after SCT fMRI MoCo (left). Group statistics for tSNR after motion correction performed either with AFNI or SCT (right). (TIF) [file pone.0320188.s001.tif]

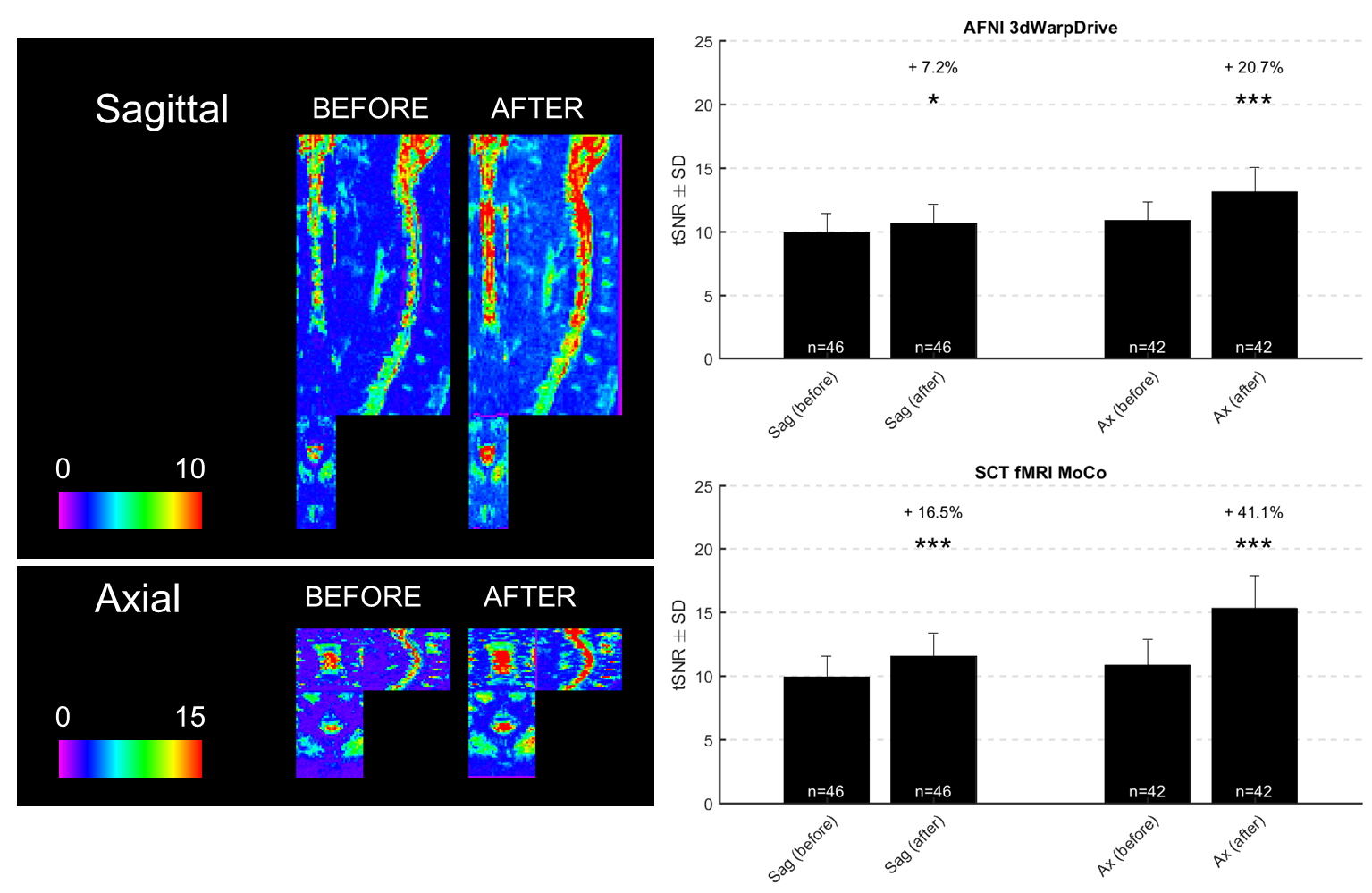

Supplement: S2 Fig — Inter-subject correlation matrices of unthresholded t map in spinal cord cord (A), including gray matter (GM) and surrounding white matter, and in central gray matter (B) are used to assess reproducibility and are shown for axial and sagittal planes. (TIF) [file pone.0320188.s002.tif]
